# Our Weights

**Published:** 1873-10

**Authors:** 


### OUR WEIGHTS.

Upon the average, boys at birth weigh a little more, and girls a little less, than six pounds and a half. For the first twelve years the two sexes continue nearly equal in weight, but beyond that time males acquire a decided preponderance. Thus, young men of twenty average about 143 lbs. each, while the young women of twenty average 120 lbs. Men reach their heaviest bulk at about thirty five, when they average about 152 lbs. ; but women slowly increase in weight until fifty, when their aveiage is about 128 lbs. Taking men and women together, their weight at full growth averages about twenty times as heavy as they were on the first day of their existence. Men range from 108 to 220 lbs., and women from 88 to 207 lbs. The actual weight of human nature, taking the average of ages and conditions—nobles, clergy, tinkers, tailors, maidens, boys, girls and babies, all included—is very nearly 100 lbs. These figures are given in avordupois weight; but the advocates of the superiority of women might make a nice point by introducing the rule that women be weighed by Troy weight—like other jewels—and men by avoirdupois. The figures will then stand: young men of twenty, 143 lbs. each; young women of twenty, 160 lbs. each; and so on.—*Sanatarian.*

